# Ischiorectal abscess with fistulization into the thigh, the ‘perfect storm’ patient

**DOI:** 10.1093/jscr/rjag840

**Published:** 2026-09-22

**Authors:** Konstantina Kostara, Erica Kozorosky, Shaban Gheith, Constantine Baltzis, Vasileios Kostaras

**Affiliations:** Department of Surgery, Hudson Regional Health, Bayonne University Hospital, 29 E 29th St, Bayonne, NJ 07002, United States; Department of Surgery, Hudson Regional Health, Bayonne University Hospital, 29 E 29th St, Bayonne, NJ 07002, United States; Department of Anatomical Sciences, St. George’s University School of Medicine, University Centre Grenada, West Indies, Grenada; Department of Emergency Medical Services, Jersey City Medical Center, 355 Grand St, Jersey City, NJ 07302, United States; Department of Surgery, Hudson Regional Health, Secaucus University Hospital, 55 Meadowlands Pkwy, Secaucus, NJ 07094, United States

**Keywords:** immunocompromised state, colonic stricture, pelvic radiation, cryptoglandular infection, ischiorectal abscess, fistula

## Abstract

Ischiorectal abscesses are a common encounter, both for general surgeons and for colorectal specialists. The formation of an ischiorectal fistula into the posterior thigh is an atypical extension that is rarely reported. In this case, the patient’s HIV history and prior chemoradiation for anal squamous cell carcinoma contributed to this unique presentation. This case highlights the importance of heightened clinical suspicion, thorough imaging, and individualized operative management in high-risk patients presenting with a seemingly straight-forward pathology.

## Introduction

Ischiorectal abscesses are deep anorectal infections that arise from obstruction of the anal glands. The majority of these abscesses follow predictable cryptoglandular pathways confined to the perianal region, and atypical extensions are exceedingly rare. Posterior thigh fistulization is an uncommon presentation, with only isolated cases described in the literature. Immunosuppression and prior pelvic radiation are well-established risk factors for severe anorectal infections, impaired wound healing, and aberrant fistula formation due to compromised local immunity and tissue damage [1, 2]. This case report highlights the combined impact of risk factors in permitting an ischiorectal abscess to dissect along nontraditional fascial planes into the posterior thigh, underscoring the importance of recognizing rare patterns of spread in high-risk patients.

## Case report

This is the case of a 46-year-old man with HIV who presented with 3 weeks of severe right gluteal pain. His history was significant for active tobacco use, anal squamous cell carcinoma treated with chemoradiation (Nigro protocol) in 2019, and large bowel obstruction secondary to a distal sigmoid stricture requiring low anterior resection in 2020. Reversal was complicated by recurrent stricture and baseline overflow fecal incontinence. At the time of presentation, the patient was compliant with HAART, with a normal CD4 count and undetectable viral load. Computed tomography (CT) demonstrated a 2.7 × 5.5 cm peripherally enhancing collection with air foci in the right ischioanal fossa and presacral region extending into the right gluteal musculature, consistent with abscess. He underwent incision and drainage and was discharged with oral antibiotics and wound care instructions.

Ten days later, he returned with persistent pain despite compliance with treatment. Repeat CT showed a 4 × 2.7 cm collection in the medial posterior gluteal soft tissue, multiple foci of air extending from the right perirectal region, and rectal wall thickening concerning for fistulous disease. He was afebrile with leukocytosis of 18.1 × 10^9^/L. Pelvic MRI confirmed persistent infection but was equivocal for a fistula. Repeat operative drainage revealed feculent material and a large cavity communicating between the rectum, right ischiorectal fossa, posterior anal region, presacral space, and posterior thigh.

**Figure 1:**
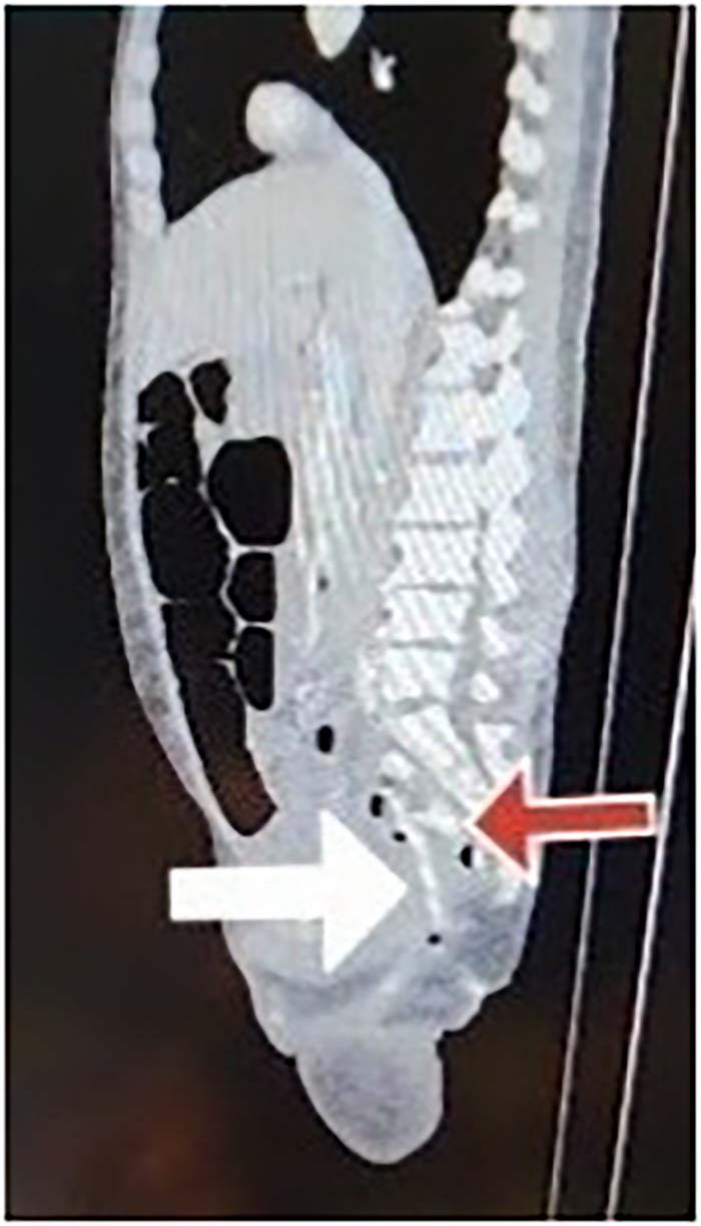
Sagittal view of ischiorectal fistula as seen on CT abdomen pelvis. Big arrow: foley catheter inserted into rectum. Small arrow: contrast blush identifying location of fistulous tract.

**Figure 2:**
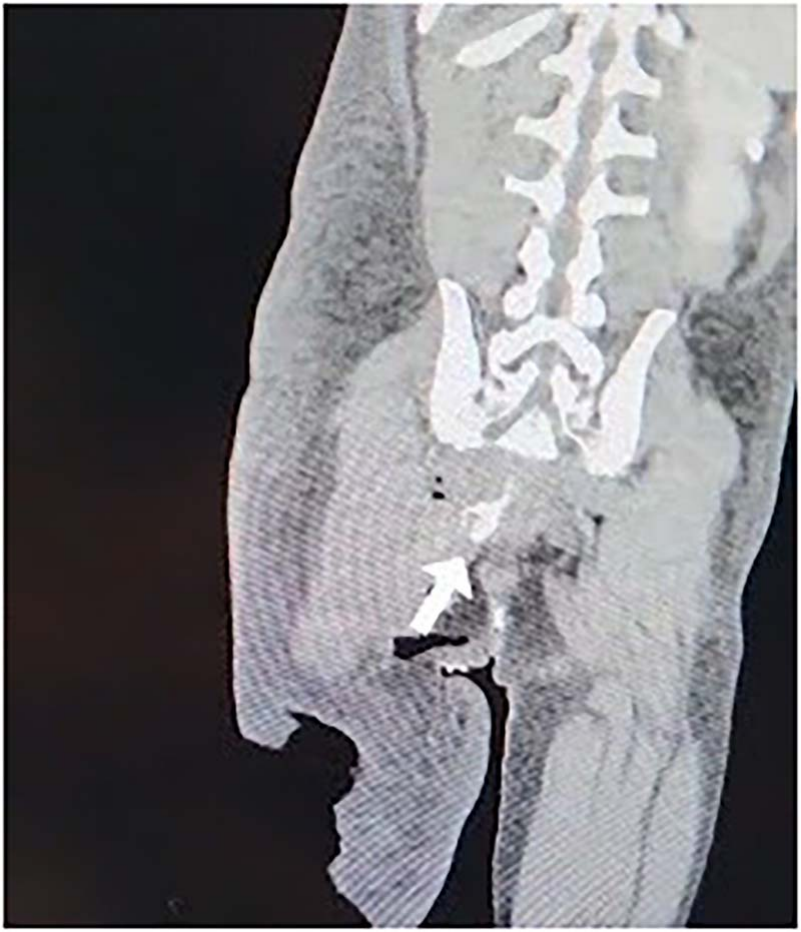
Coronal view of ischiorectal fistula as seen on CT abdomen and pelvis. Arrow: contrast blush identifying location of fistulous tract.

**Figure 3:**
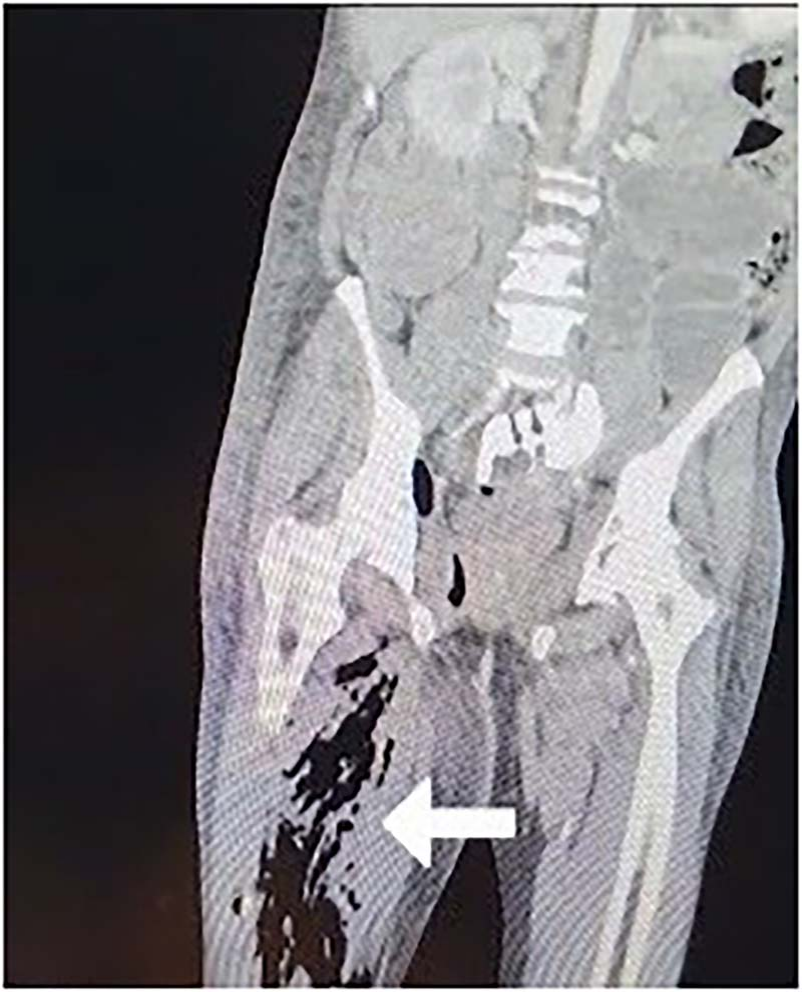
Coronal view showing extension of ischiorectal fistula into thigh compartments as seen on CT pelvis and lower extremity. Arrow: intramuscular gas and fluid tracking along the fascial planes.

CT with rectal contrast demonstrated contrast extravasation from the rectum into the right ischiorectal fossa with subcutaneous emphysema extending into the posterior thigh (Figs 1–3). The following day, the patient underwent end sigmoid colostomy creation, extensive debridement, pulse lavage, and placement of negative-pressure wound therapy. He was discharged with visiting nurse services and remains under evaluation for definitive fistula management and possible colostomy reversal.

## Discussion

The development of an ischiorectal abscess fistulizing into the posterior thigh can be primarily attributed to both the patient's HIV status and previous chemoradiation [2, 3]. The patient’s prior history of strictures and chronic constipation with overflow incontinence further added to the risk [3, 4]. Combined, these factors created a high-risk environment for development of this complex fistula.

Ischiorectal abscesses arise from cryptoglandular infection in which bacteria invade the anal glands in the Crypts of Morgagni. When the infection breaches the external sphincter, it can track laterally into the ischiorectal fossa [5, 6]. Fistula formation is a consequence of chronic infection and epithelialization of the abscess drainage tract [7]. The majority of fistulas follow predictable anatomical routes. However, in rare cases, especially when drainage is delayed or inadequate, immunosuppression is present, or extensive tissue destruction occurs, the infection may track along the loose areolar tissue and fascial planes of the ischiorectal fossa [8]. Extension of a fistula into the thigh is extremely rare and would require the infection to dissect along fascial planes beyond the typical anatomical boundaries, following the planes between the obturator internus and gluteus maximus, and then dissect inferiorly into the proximal thigh [8].

Atypical extensions are not described in the Parks classification, and there is no established standard of care for such cases. Diagnosis relies on clinical examination and imaging, with MRI being the gold standard for delineating the anatomy and extent of the fistula [6]. Treatment begins with prompt surgical drainage of any abscess, while preserving continence. Surgical options include fistulotomy (for low/simple tracts), seton placement (especially for complex or high tracts), endorectal advancement flap, ligation of the intersphincteric fistula tract (LIFT), and newer techniques such as video-assisted anal fistula treatment (VAAFT) [6, 9].

There are no established guidelines regarding the management of complex fistulas. We must place a heavy focus on prevention, aiming for early recognition and prompt surgical drainage of anorectal abscesses to prevent chronic infection and the formation of fistulas [6]. Optimizing HIV management, counseling on modifiable risk factors such as smoking and nutrition, along with multidisciplinary care is critical for improving wound healing [10]. In this case, management was guided by a staged approach, focusing first on source control with aggressive debridement and washout, followed by fecal diversion to eliminate ongoing wound contamination. Given the degree of soft tissue involvement, negative pressure vacuum therapy was initiated to promote tissue granulation within the cavity. The patient’s prior history of radiation therapy, multiple colorectal surgeries, and baseline sphincter dysfunction further complicated tissue integrity and healing capacity, reinforcing the need for a conservative, staged approach. Definitive management will depend on assessment of fistula on follow-up examinations, along with determining the success of a colostomy reversal. A multidisciplinary approach with plastic surgery, gastroenterology, and colorectal surgery will be required [11].

## Conclusion

The formation of an ischiorectal fistula into the thigh is a pattern that is rarely reported. In this case, the complex fistula formed as a result of the patient’s HIV history and prior chemoradiation. This case highlights the importance of heightened clinical suspicion, thorough imaging, and individualized management in high-risk patients presenting with anorectal pathology.

## References

[1] Vos D, Wang M, Ramaiya S et al. Anorectal pathology in the HIV population: a guide for radiologists. Abdom Radiol (NY) 2022;47:1762–74. 10.1007/s00261-022-03470-z 35284963

[2] Doyen J, Benezery K, Follana P et al. Predictive factors for early and late local toxicities in anal cancer treated by radiotherapy in combination with or without chemotherapy. Dis Colon Rectum 2013;56:1125–33. 10.1097/DCR.0b013e3182a226bd 24022529

[3] Fraunholz IB, Haberl A, Klauke S et al. Long-term effects of chemoradiotherapy for anal cancer in patients with HIV infection: oncological outcomes, immunological status, and the clinical course of the HIV disease. Dis Colon Rectum 2014;57:423–31. 10.1097/DCR.0000000000000057 24608297

[4] Sahnan K, Askari A, Adegbola SO et al. Natural history of anorectal sepsis. Br J Surg 2017;104:1857–65. 10.1002/bjs.10614 28857130

[5] Gupta AA, Gupta AM, Deshpande S et al. Treading the unusual path: a rare case of fistula-in-ano extending up to thigh. J Surg Case Rep 2025;2025:rjaf172. 10.1093/jscr/rjaf172 PMC11967179 40181925

[6] Gaertner W, Burgess PL, Davids JS et al. The American Society of Colon and Rectal Surgeons clinical practice guidelines for the management of anorectal abscess, fistula-in-ano, and rectovaginal fistula. Diseases of the Colon & Rectum 2022;65:964–85. 10.1097/DCR.0000000000002473 35732009

[7] Expert Panel on Gastrointestinal Imaging, Levy AD, Liu PS et al. ACR appropriateness criteria® anorectal disease. J Am Coll Radiol 2021;18:S268–82. 10.1016/j.jacr.2021.08.009 34794588

[8] Zhang JF, Du ML, Sui HJ et al. Investigation of the ischioanal fossa: application to abscess spread. Clin Anat 2017;30:1029–33. 10.1002/ca.22901 28509338

[9] Göttgens KW, Smeets RR, Stassen LP et al. Systematic review and meta-analysis of surgical interventions for high cryptoglandular perianal fistula. Int J Color Dis 2015;30:583–93. 10.1007/s00384-014-2091-8 25487858

[10] Pescatori M . Surgery for anal fistulae: state of the art. Int J Color Dis 2021;36:2071–9. 10.1007/s00384-021-03917-7 34057576

[11] Koutroubakis IE . The patient with persistent perianal fistulae. Best Pract Res Clin Gastroenterol 2007;21:503–18. 10.1016/j.bpg.2007.04.002 17544114

